# Species diversity and distribution of schistosome intermediate snail hosts in The Gambia

**DOI:** 10.1371/journal.pntd.0009823

**Published:** 2021-10-04

**Authors:** Ebrima Joof, Bakary Sanneh, Sana M. Sambou, Christopher M. Wade

**Affiliations:** 1 School of Life Sciences, University of Nottingham, Nottingham, United Kingdom; 2 National Public Health Laboratories, Ministry of Health and Social Welfare, Banjul, The Gambia; 3 Epidemiology and Disease Control Department, Ministry of Health and Social Welfare, Banjul, The Gambia; Stanford University Hopkins Marine Station, UNITED STATES

## Abstract

There is a need for recent information on intermediate snail hosts of schistosomes in The Gambia; the previous studies were conducted over three decades ago. This study assessed the incidence, species diversity, distribution and infection status of schistosome intermediate snail hosts in the country. Malacological surveys were conducted in all 5 regions of The Gambia: Central River Region (CRR), Upper River Region (URR), Western Region (WR), Lower River Region (LRR) and North Bank Region (NBR). Sampling of snails was undertaken at 114 sites that included permanent water bodies such as streams (bolongs), rice fields, irrigation canals and swamps; and temporal (seasonal) laterite pools. Ecological and physicochemical factors of sites were recorded. Snails were identified morphologically and screened for schistosome infections using molecular techniques. Freshwater snails were found at more than 50% (60/114) of sites sampled. While three species of *Bulinus* were collected, no *Biomphalaria* snails were found in any of the sites sampled. Of the total 2877 *Bulinus* snails collected, 75.9% were identified as *Bulinus senegalensis*, 20.9% as *Bulinus forskalii* and 3.2% as *Bulinus truncatus*. Seasonal pools produced the largest number of snails, and CRR was the region with the largest number of snails. *Bulinus senegalensis* was found more in seasonal pools as opposed to permanent sites, where *B*. *forskalii* and *B*. *truncatus* were observed to thrive. *Bulinus* snails were more common in seasonal sites where aquatic vegetation was present. In permanent sites, the abundance of snails increased with increase in water temperature and decrease in water pH. *Bulinus senegalensis* was found infected with both *S*. *haematobium* and *S*. *bovis*, while *B*. *forskalii* and B. *truncatus* had only *S*. *bovis* infection. While the human parasite *S*. *haematobium* was restricted to just four sites, the livestock parasite *S*. *bovis* had a much more widespread geographical distribution across both CRR and URR. This new information on the distribution of intermediate snail hosts of schistosomes in The Gambia will be vital for the national schistosomiasis control initiative.

## Author summary

Several studies were conducted on intermediate snail hosts of schistosomes in The Gambia between the 1950s and 1980s, but there are few reports of similar studies in recent times. After The Gambia initiated its national control programme for schistosomiasis in 2014, the need arose for more and current information on schistosomiasis and the intermediate snail hosts involved indirectly in its transmission. We undertook a malacological survey of freshwater sites in all five regions (CRR, URR, WR, LRR and NBR) of The Gambia. While no *Biomplalaria* snails were found in any of the 114 sites sampled, three species of *Bulinus* snails were found. Of the total 2877 *Bulinus* snails collected, 75.9% were identified as *Bulinus senegalensis*, 20.9% as *Bulinus forskalii* and 3.2% as *Bulinus truncatus*. CRR had the highest number of snails amongst the regions and seasonal pools yielded the most snails amongst the habitat types sampled. Habitat type, water temperature and pH of sites were observed to have an influence on snail abundance. All three *Bulinus* snails were found infected with schistosome parasites. The human parasite (*Schistosoma haematobium*) had a much more restricted distribution as opposed to the livestock parasite (*Schistosoma bovis*) which had a wider geographical range. The study provides the first malacological report of intermediate snail hosts of schistosomes in The Gambia in over 3 decades and will be useful to the national schistosomiasis control programme.

## Introduction

Schistosomiasis (also called bilharzia) is a parasitic disease caused by trematode worms (blood flukes) of the genus *Schistosoma*. It is the second most prevalent tropical disease after malaria and is a leading cause of morbidity in many parts of the globe, especially in Africa [1]. It is found in over 40 countries in Africa where nearly 192 million people are infected with the disease [2], making Africa the most prevalent continent in the world for schistosomiasis.

Globally, the human form of schistosomiasis is caused by six species in the genus *Schistosoma*: *Schistosoma haematobium*, *S*. *mansoni*, *S*. *japonicum*, *S*. *intercalatum*, *S*. *guineensis and S*. *mekongi* [3]. Most human infections result from three species, namely *S*. *haematobium*, *S*. *mansoni* and *S*. *japonicum*. The two most widely distributed species in Africa are *S*. *haematobium*, the causative agent of urogenital schistosomiasis and *S*. *mansoni*, which causes the intestinal form of the disease [3]. In The Gambia, urogenital schistosomiasis is endemic in the eastern regions (Central River and Upper River Regions) of the country [4–7], with very few reports on the occurrence of the intestinal form of the disease [7,8]. Transmission of urogenital schistosomiasis is most prevalent in these regions during the rainy season through seasonal laterite pools formed in cuirasse depressions [5]. A recent nation-wide study conducted in 2015 involving 10,434 students in 209 primary schools showed that urogenital schistosomiasis (*S*. *haematobium*) had a national prevalence of 4.2% and a regional prevalence of 14.2% for the Central River Region (CRR) and 9.4% for the Upper River Region (URR) [7]. Only 5 (0.05%) cases of intestinal schistosomiasis (*S*. *mansoni*) were detected among the school children sampled [7].

Schistosome parasites require intermediate freshwater snail hosts to develop to the infective larval stage for human infection. Snails of the genus *Bulinus* act as the intermediate hosts of *S*. *haematobium* while those of the genus *Biomphalaria* acts as the intermediate hosts of *S*. *mansoni*. These snails occur in most parts of the African continent [9,10]. *Bulinus globosus* (a member of the *Bulinus africanus* group), arguably the most important intermediate host of *S*. *haematobium* in Africa, is the most common and widely distributed species of the genus *Bulinus* in Africa [9,10]. *Bulinus forskalii* is also widely distributed in the African continent while *B*. *senegalensis* is mostly found in West Africa [9]. From early taxonomic literature, some *Bulinus* snails belonging to the *Bulinus africanus* group were difficult to distinguish in West Africa [9,11]. The smaller *B*. *jousseaumei* was regarded as a sub-species and regional form of *B*. *globosus* inhabiting the Northern limit of its range based on size variation observations of snails collected from across Angola to The Gambia [11]. A subsequent study by Mandahl-Barth [9] challenged this explanation and argued that the smaller *B*. *jousseaumei* is a distinct species in its own right, and he reported the occurrence of both the true *B*. *globosus* of typical form and normal large size as well as the smaller *B*. *jousseaumei* in The Gambia. *Bulinus truncatus* and *Bulinus guernei* were also reported to be separate species [12,13], until later studies confirmed them as the same species [14,15].

In addition to the human parasites *Schistosoma haematobium* and *S*. *mansoni*, there are 3 other schistosome species belonging to the *S*. *haematobium* group (*S*. *bovis*, *S*. *curassoni*, *S*. *mattheei*) in Sub-Saharan Africa that cause animal schistosomiasis in domestic livestock [16,17]. These schistosomes also use *Bulinus* snails as intermediate hosts and they overlap with *S*. *haematobium* in some endemic regions. Schistosomiasis in livestock such as cattle, sheep and goat is thought to have potential impact on animal health and productivity [17].

Detection and identification of schistosome infection in snails is an important component of schistosomiasis transmission surveillance and plays a huge part in unravelling the epidemiology of the disease in endemic regions. The cercarial shedding method in snails remains an important schistosome infection detection approach, but more rapid and direct techniques have been developed over the years and used as a complement [18] or alternative [19] to this long-standing traditional method. These range from simple approaches like the dot hybridisation and antibody detection methods [20,21] to more robust techniques such as the loop-mediated isothermal amplification (LAMP) assay and polymerase chain reaction (PCR) [22,23]. *Bulinus* snails are known to carry different schistosome species and detection of infection in these snails is even more complicated in transmission zones were more than one schistosome species overlap. Such a need necessitated the recent development of a molecular xenomonitoring PCR-based technique that can detect and differentially identify *S*. *haematobium* and *S*. *bovis* infections in *Bulinus* snails [24]. The method can further differentiate *S*. *bovis* from other *S*. *haematobium* group species such as *S*. *curassoni* [24].

In The Gambia, *Bulinus* species collected from streams and seasonal pools have been reported to harbour *S*. *haematobium* and *S*. *bovis* [13]. In seasonal pools, the snails involved in the transmission of *S*. *haematobium* and *S*. *bovis* were initially identified as *Bulinus forskalii* [12], but their species status was later confirmed as *Bulinus senegalensis* by Smithers [13]. Smithers [13] also reported *Bulinus jousseaumei* and *Bulinus guernei* in permanent streams. These snails were found infected with *S*. *haematobium*. *Bulinus forskalii* collected in swamps was compatible to *S*. *bovis* in the laboratory [13]. It was also found breeding in sympatry with *B*. *senegalensis* in alluvial pools and irrigated rice fields in The Gambia [25]. Few studies have reported on the intermediate host of *S*. *mansoni* in The Gambia. Smithers [13] reported *Biomphalaria pfeifferi gaudi* to be a potential intermediate host of *S*. *mansoni* in The Gambia. Subsequently, in 1957, *Biomphalaria pfeifferi gaudi* snails collected from a stream were observed shedding *S*. *mansoni* cercariae and intestinal schistosomiasis infections were confirmed in inhabitants of an adjacent village, Jiboroh in the Western Region of The Gambia [8]. Later studies conducted in the 1980s on schistosomes and their intermediate snail hosts in The Gambia progressed from observational to interventional strategies with mollusciciding (with niclosamide) used in a study to control *B*. *senegalensis* populations in seasonal pools [26]. *Bulinus senegalensis* populations were progressively reduced to almost 1% of the numbers in untreated pools and there was no evidence of new infection in children from the intervention area for a period of three years [26].

After the London declaration of 30^th^ January 2012 [27], which had advocated for the elimination of schistosomiasis in some countries, the move to control schistosomiasis and other neglected tropical diseases (NTDs) has intensified in many African countries. To meet the global objective of combating NTDs, The Gambia started the process of developing a national control programme for these diseases in 2014. A situation analysis was conducted from May to June 2014 to assess the status of NTDs in the country. This review based on published data and routine hospital reports revealed the endemicity of schistosomiasis in the country and established schistosomiasis (along with soil transmitted helminthiasis) as the main NTDs requiring a control program in the country. It further revealed knowledge gaps on the status of schistosomiasis infection in the country [28] that led to a nationwide mapping of schistosomiasis from May to June 2015. Based on the prevalence revealed by the 2015 study [7], a mass drug administration campaign for schistosomiasis control was conducted in March 2017 in 19 districts [29]. According to WHO guidelines for the control of schistosomiasis, the next interventions that should follow should focus on the intermediate snail hosts, as well as on behaviour, water, sanitation and hygiene. Since studies on snail intermediate hosts of schistosomes in The Gambia have been very limited, and the latest reported studies, covering just a few areas of the country, were conducted in the 1980s [26], there is need for a reliable, up to date knowledge on the distribution of schistosome intermediate snail hosts in a wider geographical scope, to effectively guide the national schistosomiasis control programme of the country.

This study therefore aimed to assess the current incidence, species diversity, distribution and infection status of potential intermediate snail hosts of schistosomes in The Gambia. The study sought to address the following specific research questions: 1) which intermediate snail hosts of schistosomes are most abundant and widely distributed? and 2) which habitat types and regions have the highest number of intermediate snail hosts?

## Methods

### Study area and site selection

**Fig 1:**
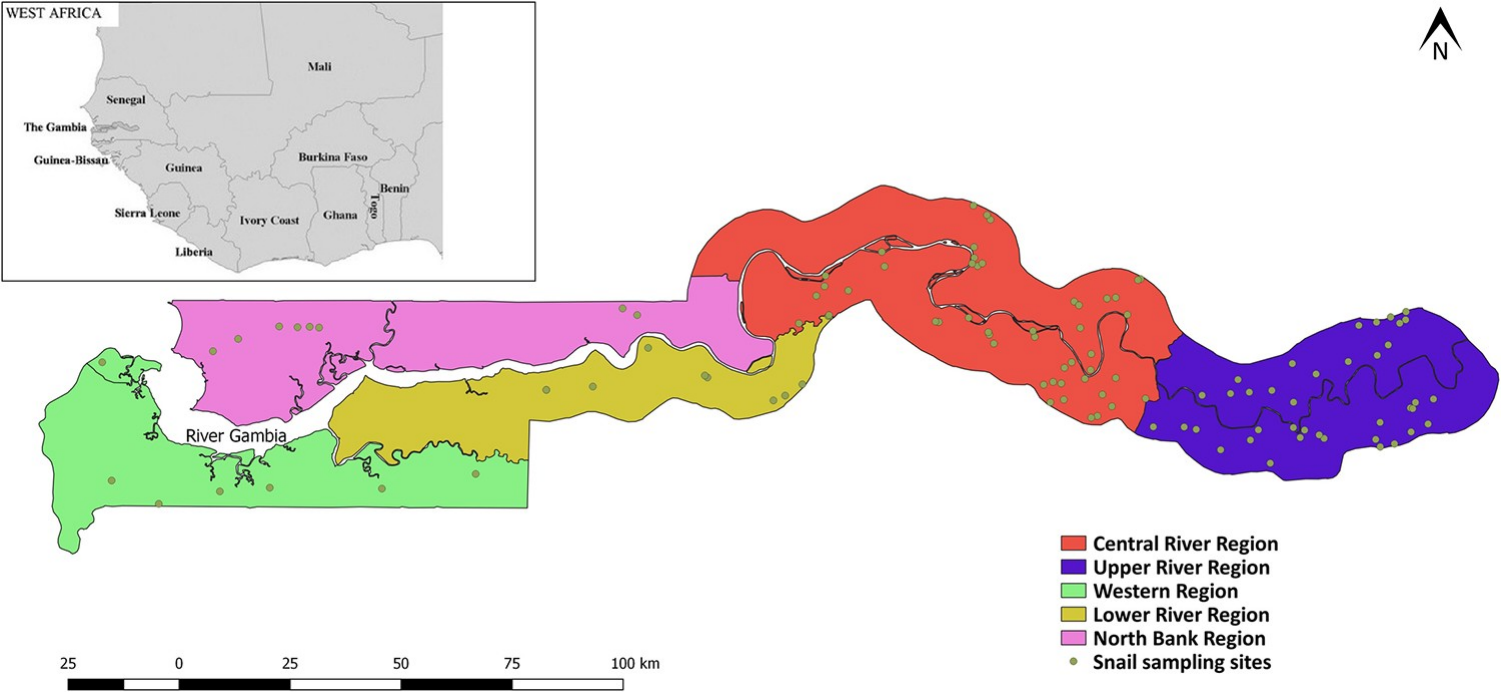
A map of The Gambia showing the snail sampling sites within the 5 regions of the country. Link to base layer of map used in the figure: https://data.humdata.org/dataset/gambia-administrative-boundaries.

**Fig 2:**
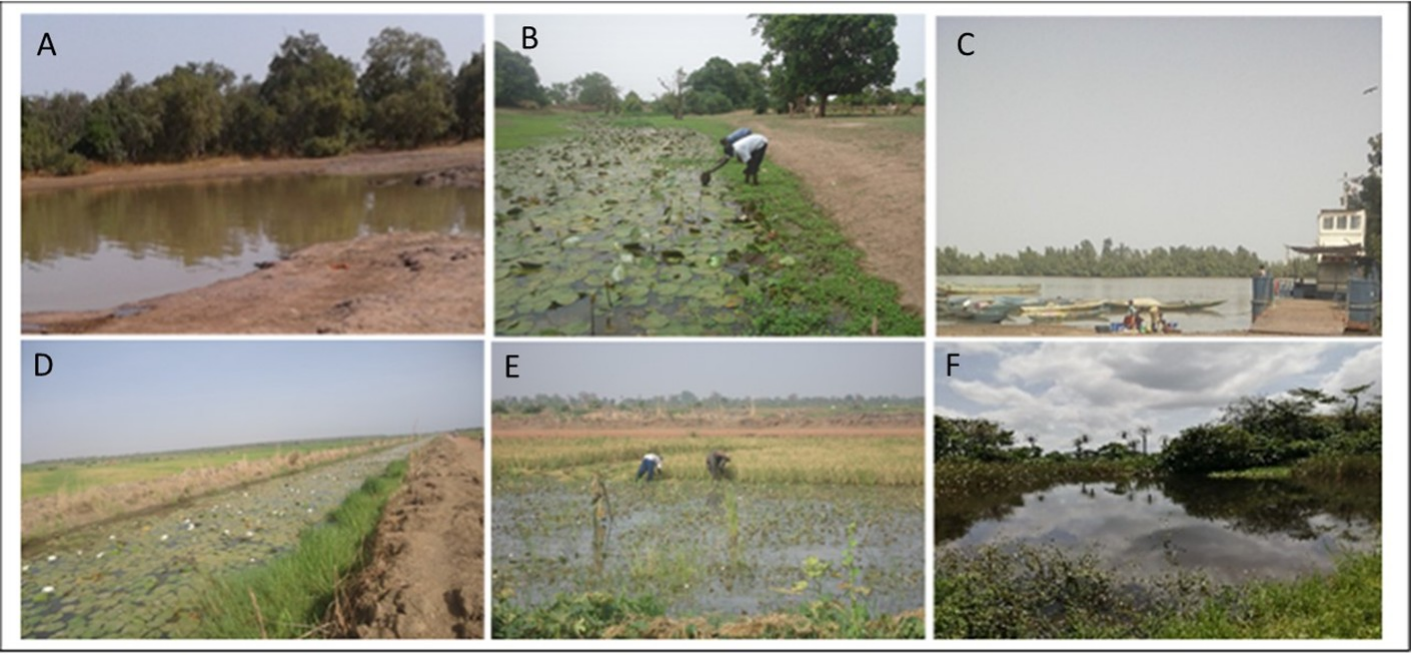
Different types of habitats from which snails were sampled; A–seasonal laterite pool, B–stream, C–river, D–irrigation canal, E–rice field, F–swamp.

Malacological surveys were conducted in all 5 regions of The Gambia: Central River Region (CRR), Upper River Region (URR), Western Region (WR), Lower River Region (LRR) and North Bank Region (NBR). Sampling was done at 114 sites: 50 sites in CRR, 40 sites in URR, 7 sites in WR, 9 sites in LRR and 8 sites in NBR (Fig 1). Communities that were found to have human schistosomiasis according to recent previous studies [7,30] were selected for sampling. These communities were visited, and the inhabitants asked about the location of freshwater sites and information on human and livestock water contact at these sites. Several other communities that did not have human schistosomiasis according to the previous studies were also selected if they had freshwater sites that were frequented by humans or livestock. The identified freshwater sites in these communities were visited and sampled. Types of habitats sampled were permanent water bodies that included streams (bolongs), rice fields, irrigation canals and swamps; and temporal (seasonal) laterite pools (Fig 2).

### Snail sampling and processing

Sampling of Snails was undertaken from 114 sites across The Gambia in 2017 and 2019. The 2017 collections were conducted in June in permanent sites and from October to November in seasonal sites. In 2019, the collections were undertaken in March in permanent sites and from September to October in seasonal pools. Sampling from permanent sites was carried out during the dry season because this is the time when snails are abundant at these sites. They are very scarce or non-existent during the rains due to the overflowing of water. Sampling of seasonal pools was conducted in the rainy season because this is the only time these bodies of water exist.

For each collection site, two field collectors searched for 30 minutes over a length of approximately 15m along the bank of streams, rivers and irrigation canals, and an area of approximately 5m^2^ for seasonal pools, rice fields and swamps. Snails found were collected with a scoop, and those attached to vegetation and sticks were handpicked, as some snails attach to the underside of aquatic vegetation. The habitat type, site location, date and time of sampling and geographical coordinates (using a GARMIN Oregon 550 GPS device) of each site were recorded. Collected snails were placed in well labelled separate containers for each site and transported to the Microbiology Laboratory of Bansang General Hospital in CRR. In the laboratory, snails were sorted, identified, and counted. The collected snails were identified based on morphological characteristics of their shells [10,31]. *Bulinus forskalii* was differentiated from *Bulinus senegalensis* by the presence of a distinctive shoulder angle on the upper whorls (as opposed to more round and smooth upper whorls in *B*. *senegalensis*) [31]. However, this feature is not easily identifiable in some snails and the species identification of such snails was confirmed by molecular barcoding using cytochrome oxidase I primers LCO: 5’- GGTCAACAAATCATAAAGATATTGG-3’ and HCO: 5’- TAAACTTCAGGGTGACCAAAAAATCA-3’ as described in Folmer et al [32]. Furthermore, to confirm the accuracy of our morphological identifications, the identification of 10% of our samples was confirmed by molecular methods. This confirmed that in all cases our morphological identifications were accurate.

### Infection screening of *Bulinus* snails

For each site, 20 *Bulinus* snails were selected at random and screened for schistosome infection using a molecular detection method. For sites with fewer than 20 *Bulinus* snails, all individuals were examined. DNA was extracted from the snails using the CTAB DNA extraction protocol as described in Joof et al [33]. A forward *Schistosoma*-specific primer (ITS2_Schisto_F: 5’-GGAAACCAATGTATGGGATTATTG-3’) [34] and a universal reverse primer (ETTS1: 5’-TGCTTAAGTTCAGCGGG-3’) [35] were utilised in a PCR as described by Pennance and colleagues [24]. The combination of these primers amplifies a 538bp fragment of the ITS2 gene of *Schistosoma* DNA which can be sequenced to detect and differentially identify *S*. *haematobium* and *S*. *bovis* infections in *Bulinus* snails. PCR reactions were carried out in a final volume of 25μl containing 2.5 μl 10x reaction buffer, 1.25μl MgCl_2_ (2.5mM), 4 μl pre-mixed dNTPs (0.2mM), 0.5 μl of each primer (0.2μM) and 1 unit of Taq polymerase (Bioline), and 1 μl of template DNA from snail extract. The PCR cycling conditions were as follows: pre-heating at 95°C for 5 minutes, followed by 40 cycles at 95°C for 30 seconds, 58°C for 30 seconds and 72°C for 90 seconds, then a final extension at 72°C for 10 minutes.

For the visualisation of PCR results, 5μL of each PCR product was run on a 1.5% agarose gel (stained with ethidium bromide) for 1 hour at 100 V. Band sizes of amplicons were viewed using NuGenius gel documentation system (Syngene, Cambridge, UK). The PCR products were Sanger sequenced using the Macrogen Eco-sequencing service (Macrogen B.V, Amsterdam, Netherlands). The sequence data were managed using the STADEN package v.1.5.3 [36] and the Genetic Data Environment (GDE) package v.2.2 [37]. Sequences were then compared with *S*. *haematobium* and *S*. *bovis* reference sequences and the four single nucleotide polymorphism (SNP) sites in the ITS2 region of the two species used to confirm the species identification of each sequence. The samples that matched *S*. *bovis* reference sequences were further processed by amplifying the ITS1 region of *Schistosoma* in these samples using primers ETTS2: 5’-TAACAAGGTTTCCGTAGGTGA-3’ and ITS2_Schisto_R: 5’-ATTAAGCCACGACTCGAGCA-3’ [24] to confirm if they are *S*. *bovis* or *S*. *curassoni*. *Schistosoma bovis* and *S*. *curassoni* have the same nucleotide bases at the four SNP sites in the ITS2 region but differ at the lone SNP site in the ITS1 region of *Schistosoma* where they can be differentially identified.

### Ecological and physicochemical parameters

For the 2017 collections, presence or absence of aquatic vegetation and algae were recorded. The water temperature and water pH of the collection sites were taken using a digital probe thermometer 31/162/0 (Brannan Thermometers, Cumbria, England) and a Mettler Toledo EL20 pH meter (Fisher Scientific, Loughborough, UK)

### Data processing and analysis

Raw data on snails collected per site were entered on Microsoft Excel and all statistical analyses were carried out using R Version 4.1.0 [38]. Differences in snail abundance among *Bulinus* species, regions and habitat types were statistically compared using Kruskal-Wallis and Wilcoxon (pairwise) Rank Sum tests, and depicted using box and whisker plots. Maps showing the distribution of *Bulinus* snails and schistosome parasites were created using QGIS version 3.0 software (Girona Open Source Geospatial Foundation). Negative binomial generalised linear mixed models (GLMMs) were fitted in R Version 4.1.0 using the package ‘glmmTMB’ [39] to test for associations between the abundance of *Bulinus* snails and environmental/physicochemical parameters. Variance inflation factor (VIF) was used to determine the relationships and collinearity between variables. A VIF value of more than 5 was indicative of collinearity [40]. We used the Akaike’s Information Criterion (AIC) and negative log-likelihood values to compare models and choose the final models. The regression analyses were done separately for seasonal sites and permanent sites.

## Results

### Presence and incidence of potential schistosome snail hosts

A total of 114 sites were visited. Snails were found at 60 of these sites, while no snails were found at 54 of the sites. *Bulinus* snails were found at 59 sites. No *Biomphalaria* snails were observed at any of the 114 sites sampled. In addition to intermediate snail hosts of schistosomes, other snails (some of which are intermediate hosts of other parasites) found in these freshwater habitats included *Radix natalensis*, *Gyralus spp*. and *Lanistes spp*. One hundred and sixty-five *Radix natalensis* were recovered from three permanent sites, 120 *Gyralus spp*. were collected from 9 permanent sites, and 8 *Lanistes spp*. were found in one seasonal and two permanent sites (S1 Table).

### Species diversity, distribution and abundance of *Bulinus* snails

**Fig 3:**
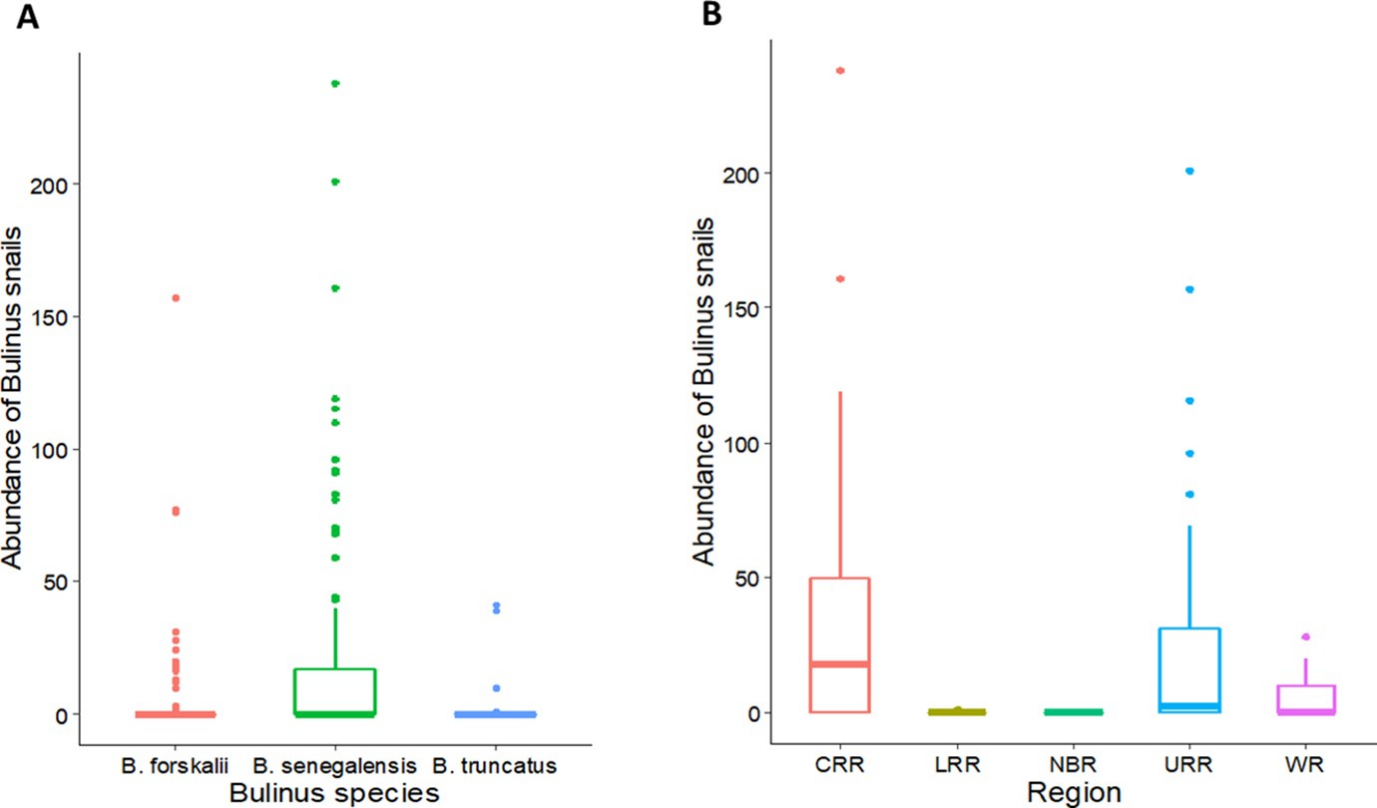
Box and Whisker plots showing the abundance of *Bulinus* snails by: (A) species and (B) region. The box represents the lower quartile (0.25), the median (0.5) and the upper quartile (0.75); the whiskers depict variability outside the lower and upper quartiles. Outliers are shown as individual points.

A total of 2877 *Bulinus* snails comprising three species were collected in the study. *Bulinus senegalensis* was the most common species making up 75.9% (2184) of all *Bulinus* snails, followed by *Bulinus forskalii* with 20.9% (602), then *Bulinus truncatus* with 3.2% (91) (Fig 3). There was a significant difference in abundance among the three *Bulinus* species collected (Kruskal-Wallis χ 2 = 41.592, df = 2, p < 0.001) with the number of *B*. *senegalensis* significantly higher than both *B*. *forskalii* (p < 0.001) and *B*. *truncatus* (p < 0.001) and *B*. *forskalii* significantly higher than *B*. *truncatus* (p < 0.001). Abundance of *Bulinus* snails also differ among the regions (Kruskal-Wallis χ 2 = 19.567, df = 4, p = 0.001) with CRR having the largest number of *Bulinus* snails, followed by URR, WR, LRR, and then NBR (Fig 3). CRR had significantly more *Bulinu*s snails than both LRR (p = 0.012) and NBR (p = 0.012). URR also had significantly more snails than both LRR (p = 0.024) and NBR (p = 0.021).

**Fig 4:**
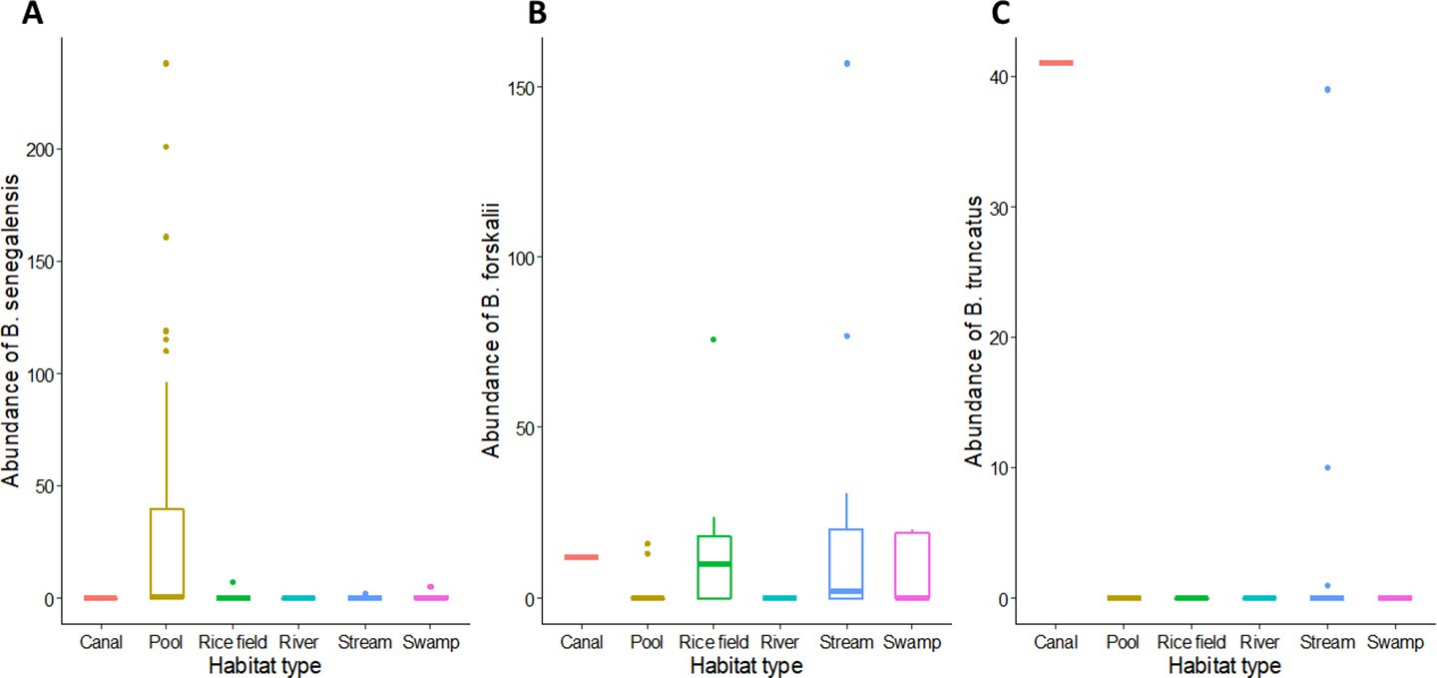
Box and Whisker plots showing the abundance by habitat type of *Bulinus* snails: (A) *B*. *senegalensis*, (B) *B*. *forskalii* and (C) *B*. *truncatus*. The box represents the lower quartile (0.25), the median (0.5) and the upper quartile (0.75); the whiskers depict variability outside the lower and upper quartiles. Outliers are shown as individual points.

**Fig 5:**
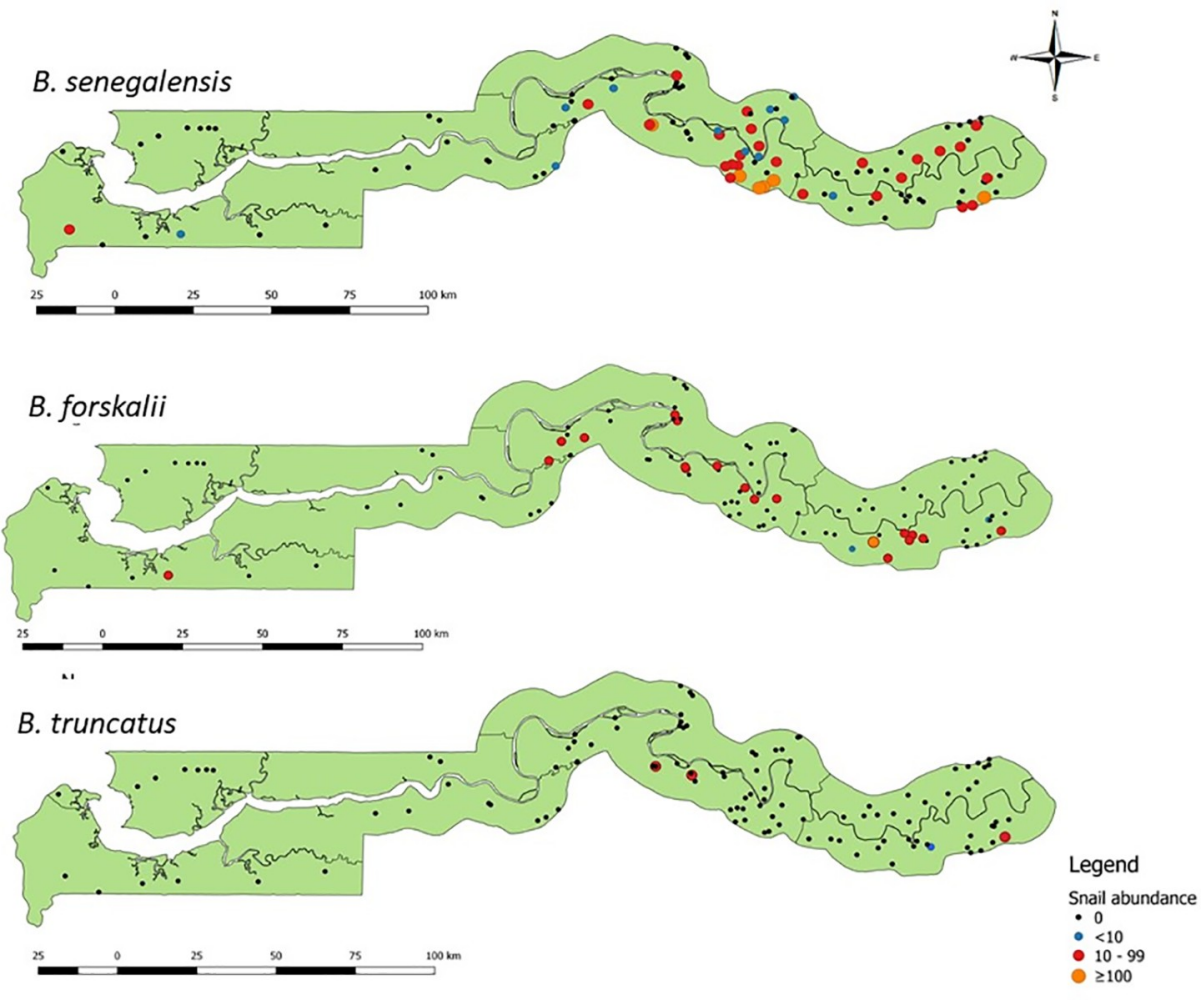
Maps of The Gambia showing the abundance and distribution of *Bulinus senegalensis*, *Bulinus forskalii*, and *Bulinus truncatus* at sampling sites across the study regions. Link to base layer of map used in the figure: https://data.humdata.org/dataset/gambia-administrative-boundaries.

*Bulinus senegalensis* was the most abundant and widespread species found in 42 (71.2%) of the 59 sites inhabited by *Bulinus* snails. It was present on either side of the River Gambia and observed in all study regions except NBR. *Bulinus senegalensis* snails were most abundant in seasonal pools (Fig 4), where they were mostly observed aggregated together. Thirty-eight of the 42 sites where *B*. *senegalensis* was observed were seasonal pools with 2169 *B*. *senegalensis* snails found in seasonal pools compared to just 2 snails in streams, 6 snails in swamps and 7 snails in rice fields. These differences in the abundance of *B*. *senegalensis* among habitat types were statistically significant (Kruskal-Wallis χ 2 = 20.015, df = 5, p = 0.001) with the number of *B*. *senegalensis* significantly higher in seasonal pools than in streams (p = 0.003). Fig 5 shows the abundance and distribution of *B*. *senegalensis* by region. Twenty-three sites in CRR yielded no *B*. *senegalensis* snails, 8 sites had <10 snails, 14 sites yielded 10 to 99 snails and 5 sites produced ≥100 *B*. *senegalensis* snails (Fig 5). In URR, 27 sites had no *B*. *senegalensis* snails, a lone site yielded <10 snails, 11 sites had 10–99 snails, and just 1 site had ≥100 *B*. *senegalensis* snails. WR had two sites that yielded *B*. *senegalensis* and LRR had one site with *B*. *senegalensis* snails. NBR sites yielded no *B*. *senegalensis* snails (See Fig 5). See S1 Table for the actual count of snails per species and per site and the GPS coordinates of the sites.

*Bulinus forskalii* was the second most common species. It was also present on both sides of the River Gambia and was collected from 35.6% (21/59) of the sites where *Bulinus snails* were found. Most of the sites (90.5%) from where this species was found were permanent sites, such as streams, swamps and rice fields (Fig 4). Twenty-nine *B*. *forskalii* were collected from seasonal pools, 382 from streams, 39 from swamps, 140 from rice fields and 12 from an irrigation canal. Abundance of *B*. *forskalii* was significantly different among the habitat types (Kruskal-Wallis χ 2 = 42.546, df = 5, p < 0.001); seasonal pools had significantly less *B*. *forskalii* than streams (p < 0.001), swamps (p = 0.001), rice fields (p < 0.001) and irrigation canals (p-value < 0.001). At most permanent sites, *B*. *forskalii* was found in small numbers and sparsely distributed, but in a few sites (Bansang in CRR and Sotuma Sire and Misra Ba Mariama in URR) where there was abundant vegetation, they were found clustered together in large numbers. In CRR, 39 sites had no *B*. *forskalii* and 11 had 10–99 *B*. *forskalii snails* (Fig 5). URR which had the largest number of *B*. *forskalii*, had 31 sites with no *B*. *forskalii* snails, 2 sites with <10 snails, 6 sites with 10–99 snails and 1 site with ≥100 *B*. *forskalii snails*. WR had just one site that had *B*. *forskalii* and neither LRR nor NBR sites had *B*. *forskalii* snails (Fig 5). See S1 Table for the actual count of snails per species and per site and the GPS coordinates of the sites.

Unlike *B*. *senegalensis* and *B*. *forskalii*, the presence and distribution of *B*. *truncatus* was limited. It was the least encountered species collected at only 4 (6.8%) sites. It was found only in permanent streams and one irrigation canal (Fig 4). Fifty *B*. *truncatus* were collected in streams and 41 from the irrigation canal. The difference in abundance of *B*. *truncatus* among habitat types was statistically significant (Kruskal-Wallis χ 2 = 39.042, df = 5, p < 0.001); even though the number of *B*. *truncatus* collected from streams was only slightly higher than the number from the irrigation canal, the difference was still statistically significant (p = 0.043). The distribution of *B*. *truncatus* was confined to the south bank of the River Gambia, and it was found only in CRR and URR. One stream and one irrigation canal in CRR yielded 10 and 41 *B*. *truncatus*, respectively. One and 39 *B*. *truncatus* were recovered from two streams in URR (Fig 5). *Bulinus truncatus* was observed attached to aquatic plants such as waterlily in all these sites. See S1 Table for the actual count of snails per species and per site and the GPS coordinates of the sites.

As much as these *Bulinus* species seemed to have preference for specific habitat types, they were sometimes found together in sympatry with other *Bulinus* species at the same site. *B*. *senegalensis* and *B*. *forskalii* were observed together at four permanent sites including a stream, swamp and rice field. The two species were also found together in two seasonal pools. *B*. *forskalii* was also observed co-existing with *B*. *truncatus* in a stream and in an irrigation canal. While *B*. *forskalii* was found in sympatry with either *B*. *senegalensis* or *B*. *truncatus*, *B*. *senegalensis* was never observed in the same habitat with *B*. *truncatus*.

### Physicochemical and environmental factors

Data on ecological factors were available for only the 2017 collections. At seasonal sites, water temperature ranged from 22.1–38.3°C and the median (interquartile range) water temperature was 29.3°C (27.8–33.1°C). The water pH of these seasonal sites ranged from 6.42–8.16 and the median (interquartile range) water pH was 6.95 (6.78–7.26). From the negative binomial regression in glmmTMB, only presence of aquatic vegetation had a statistically significant positive association with abundance of *Bulinus* snails in seasonal sites (*p*-value = 0.014). The other variables (presence of algae, water temperature and pH) did not show any statistically significant association with abundance of *Bulinus* snails in seasonal sites (Table 1).

**Table 1:** Summary of negative binomial regression (GLMM) of *Bulinus* snail abundance and environmental variables. Analyses done using glmmTMB package in R 4.1.0.

	Variables	Estimate	Std. Error	Confidence Interval (CI)	*p*-value
**Seasonal sites**	Intercept	2.668	6.625	-10.317–15.654	0.687
	Aquatic vegetation	1.565	0.637	0.317–2.813	0.014*
	Algae	-0.117	0.870	-1.822–1.587	0.893
	Temperature	-0.056	0.081	-0.215–0.102	0.484
	pH	0.268	0.670	-1.046–1.581	0.690
**Permanent sites**	Intercept	3.605	8.958	-16.829–17.039	0.967
	Aquatic vegetation	10.240	8.760	-15.807–18.287	0.906
	Algae	0.085	0.629	-1.148–1.317	0.893
	Temperature	0.250	0.118	0.018–0.482	0.034*
	pH	-2.729	0.777	-4.253 –-1.206	0.0004*

*Statistical significance at *p*<0.05

Negative binomial glmm analyses performed separately for seasonal sites and permanent sites.

For the permanent sites, water temperature ranged from 23.1–33.9°C and the median (interquartile range) water temperature was 27.6°C (25.3–30.0°C). The water pH at these permanent sites ranged from 5.52–7.55 and the median (interquartile range) water pH was 6.96 (6.37–7.17) (see S2 Table). Water temperature showed a significant positive relationship (*p*-value = 0.034) while water pH showed a significant negative relationship (*p*-value < 0.001) with abundance of *Bulinus* snails in permanent sites (Table 1). Presence of aquatic vegetation and algae did not have any significant association with abundance of *Bulinus* snails in permanent sites.

### Infection of *Bulinus* snails with schistosome parasites

Of the 883 *Bulinus* snails screened for schistosome infection, 77 (8.7%) were infected with schistosomes (either *S*. *haematobium* or *S*. *bovis*). These comprised 52 (5.9%) *B*. *senegalensis*, 19 (2.2%) *B*. *forskalii* and 6 (0.7%) *B*. *truncatus*. Both *S*. *haematobium* and *S*. *bovis* infections were observed in *B*. *senegalensis* snails with 7 (1.2%) *B*. *senegalensis* infected with *S*. *haematobium* and 45 (7.7%) infected with *S*. *bovis* (Table 2). No co-infection of *S*. *haematobium* and *S*. *bovis* was observed in *B*. *senegalensis* snails. *Bulinus forskalii* had only *S*. *bovis* infections, with 19 (7.7%) of this species found harbouring this livestock parasite. *Bulinus truncatus* also had only *S*. *bovis* infection, with 6 (12.0%) snails infected (Table 2).

**Table 2:** Infection of *Bulinus* snails with *Schistosoma haematobium* and *Schistosoma bovis*.

	*B*. *senegalensis*	*B*. *forskalii*	*B*. *truncatus*
Number of snails screened	586	247	50
No. Infected with *S*. *haematobium* (%)	7 (1.2%)	0 (0%)	0 (0%)
No. Infected with *S*. *bovis* (%)	45 (7.7%)	19 (7.7%)	6 (12.0%)
Total (%)	52 (8.9%)	19 (7.7%)	6 (12.0%)

**Fig 6:**
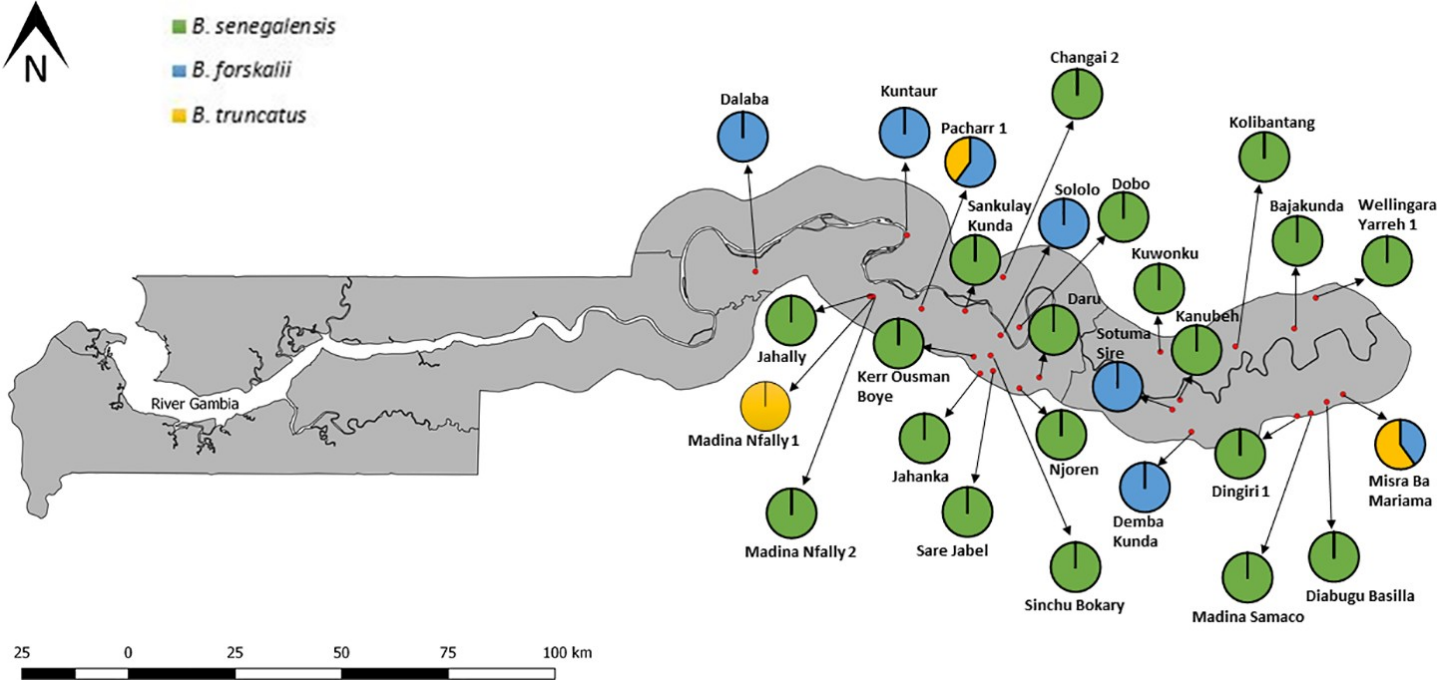
Distribution of infected *Bulinus* species in the study. Link to base layer of map used in the figure: https://data.humdata.org/dataset/gambia-administrative-boundaries.

Infected *Bulinus* snails were found at 27 sites, which included seasonal pools, streams, a swamp, ricefield and irrigation canal. Nineteen sites had infected *B*. *senegalensis*, 5 sites had infected *B*. *forskalii*, 1 site had infected *B*. *truncatus* and 2 sites had both infected *B*. *forskalii* and *B*. *truncatu*s. All these sites were in CRR and URR (Fig 6).

**Fig 7:**
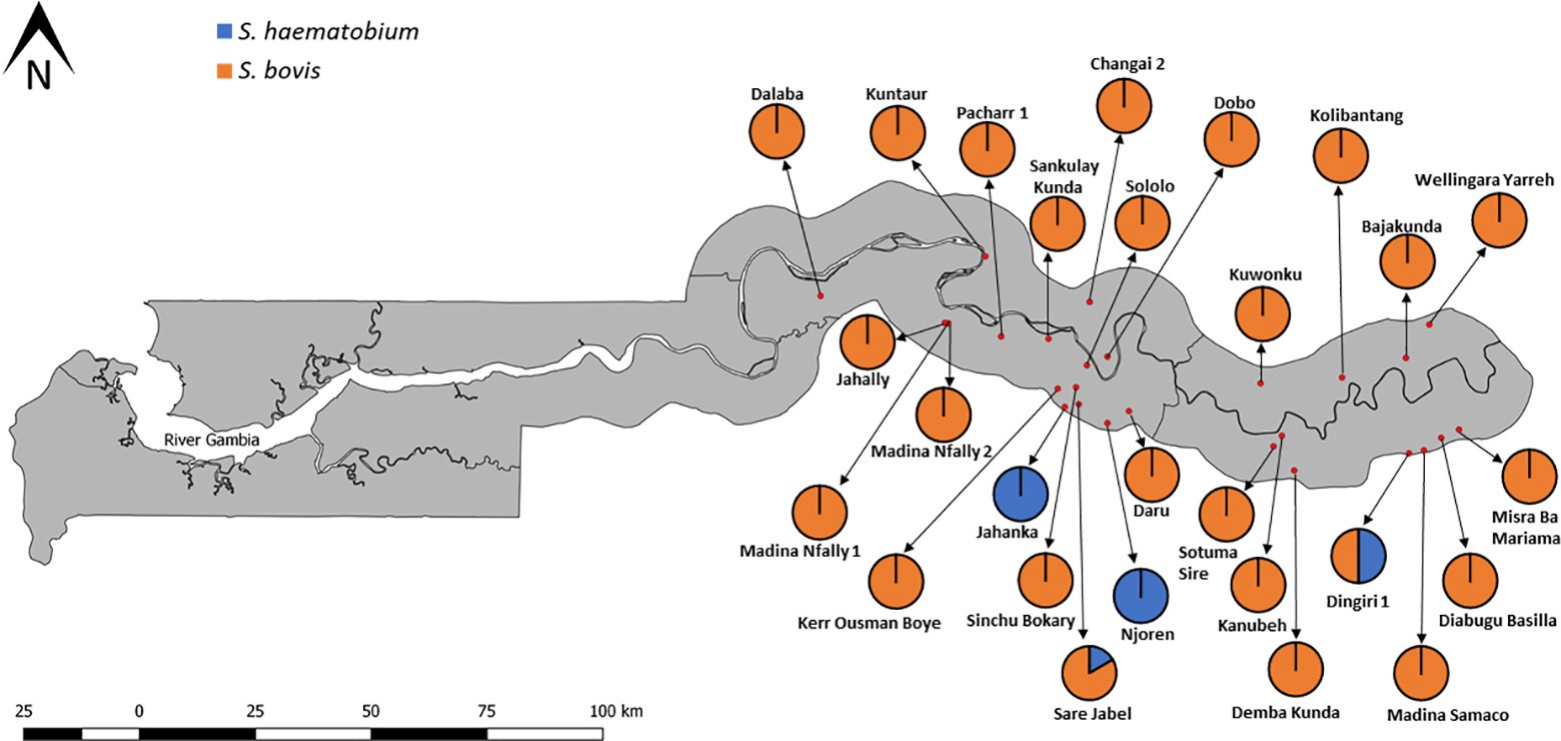
Distribution of *Schistosoma haematobium* and *Schistosoma bovis* in the study. Link to base layer of map used in the figure: https://data.humdata.org/dataset/gambia-administrative-boundaries.

*Schistosoma haematobium* was discovered in 4 study sites which were all seasonal pools and had only *B*. *senegalensis* snails, with three of the sites (Jahanka, Njoren and Sare Jabel) in CRR and 1 site (Dingiri 1) in URR (Fig 7). Jahanka and Njoren had 3 (15%) and 1 (5%) infected snails, respectively; all infections were *S*. *haemotobium*. Sare Jabel had 1 snail (5%) infected with *S*. *haematobium* and 5 snails (25%) infected with *S*. *bovis*. Dingiri 1 was the only site with *S*. *haematobium* infected snails in URR, with 2 (10%) snails infected with *S*. *haematobium* and another 2 (10%) infected with *S*. *bovis*. Only *S*. *bovis*, which is the predominant schistosome species observed in this study, occurred in the remaining 23 sites where infected snails were found, with 13 of the sites in CRR and 10 in URR (Fig 7). *Schistosoma bovis* infection rate at these sites ranged from 5% to 40% for all three *Bulinus* species (See S3 Table). *Schistosoma bovis* infection rates for CRR sites ranged from 5% to 40% and infection rates for URR sites ranged from 5% to 20%. These sites included all the habitat types in which snails were found in this study.

## Discussion

This study is the first malacological survey of intermediate snail hosts of schistosomes conducted in The Gambia in more than 30 years and is the first study to include all the regions of the country. *Bulinus* snails were found in 98% of the aquatic sites where snails were observed.

*Bulinus senegalensis* was found to be the most common and widely distributed species in The Gambia in this study. It was found on both sides of the River Gambia and in all but one region of the country. This widespread distribution of *B*. *senegalensis* is similar to what was observed in neighbouring Senegal as the snail species was found in many of its regions [41–43]. *Bulinus senegalensis* was most common in CRR and URR which share the same ‘tall-grass savanna shrub’ ecological zone with the Senegalese regions in which the snail was mostly reported [41–43]. They were mostly found in seasonal pools often in large numbers in these two regions. Away from the river in these regions lies a sandstone plateau which has a rocky, laterite iron pan (cuirasse) termed a laterite plateau [44]. Depressions formed in this plateau hold rainwater and form seasonal pools during the rainy season which become breeding grounds for *B*. *senegalensis* [5]. Despite the short (4–5 months) span of these pools *B*. *senegalensis* can continue breeding year after year in these sites due to its short reproductive cycle and its behaviour of aestivation [41,43]. African Bulinid snails are known for their ability to aestivate and survive in dried habitats during long dry periods [5,10]. In this study *B*. *senegalensis* were observed in seasonal pools in CRR and URR, even those at the verge of drying and holding little water. The other 3 regions (LRR, NBR and WR) where the laterite plateau is not found have few seasonal pools and thus few *B*. *senegalensis*. Just as has been reported in other parts of Africa [45], this study shows that seasonal rain-fed pools are important habitats of *S*. *haematobium* intermediate snail hosts. *Bulinus senegalensis* was also found in small numbers in streams and rice fields. This is consistent with the findings of previous studies undertaken in The Gambia and elsewhere [25,46].

*Bulinus forskalii* was the second most encountered species. It was also observed on both sides of the River Gambia, and found in CRR, URR and WR. The geographical range of *B*. *forskalii* has been reported to extend across many parts of Africa including Madagascar and São Tomé Island [10]. Although *B*. *forskalii* was found in two seasonal pools, it was mostly found in streams and rice fields in this study. Previous studies in Senegambia have reported the existence of *B*. *forskalii* in such habitat types [15,25]. The River Gambia is fresh along CRR and URR and its tributaries (streams) along these regions (locally called ‘bolongs’) are also freshwater. The river also provides water supply to a large irrigation scheme in CRR which is used for rice growing. These freshwater streams and irrigated rice fields provide breeding grounds for *B*. *forskalii* which proliferate more in permanent habitats [47]. *Bulinus forskalii* was more common in URR than CRR because URR has a lot more streams than CRR. WR has a single site (swamp) where *B*. *forskalii* was collected. No *B*. *forskalii* were found in sites in either LRR or NBR. Since the River Gambia is brackish along these 3 regions, the existence of freshwater streams and swamps in these regions is rare.

*Bulinus truncatus* was the least common and least distributed species in this study. It was observed in streams and in an irrigation canal in only CRR and URR. An earlier study in the 1950s reported snails from some localities in The Gambia as *Bulinus truncatus* [12], but a later study identified similar snails from these same localities as *Bulinus guernei* [13]. In later years, studies on various *Bulinus truncatus* and *Bulinus guernei* samples from different parts of Africa that utilised shell morphology, biochemical and cytological analyses found no taxonomic subdivision between the species and suggested they are the same [14,15]. *Bulinus guernei* reported in Gambia by Smithers [13] were collected from streams in URR, the same region and type of habitat in which *B*. *truncatus* was found in this study. *B*. *truncatus* was also found in CRR in this study.

*Bulinus senegalensis* was mostly found alone in seasonal pools but was occasionally observed together with *B*. *forskalii* in seasonal pools and in permanent sites (stream, swamps and rice fields). *Bulinus forskalii* also co-inhabited a single stream and a single irrigation canal with *B*. *truncatus*. Goll [25] also found *B*. *senegalensis* and *B*. *forskalii* breeding together in alluvial pools and irrigated rice fields in The Gambia.

Despite this huge incidence and presence of *Bulinus* snails, *Biomphalaria* snails were not found in any of the sites sampled. The occurrence of *Biomphalaria pfeifferi gaudi* in The Gambia was reported in the 1950s in just two studies [8,31] but numerous studies conducted on freshwater snails in the country between the 1950s and 1980s have failed to identify *Biomphalaria* in The Gambia [5,9,12,25,26]. This corresponded with very few reports on the existence of intestinal schistosomiasis among the human population [8]. In more recent times, there were few or no reports of intestinal schistosomiasis cases in health facilities across the country as opposed to the urogenital form of the disease [28], which is consistent with findings of recent prevalence studies of the disease [6,7].

*Bulinus* snails were observed to be more common at seasonal sites where aquatic vegetation such as water lilies and ‘Mandinka rice’ were present. Some previous studies have found snails to be more abundant at sites where aquatic vegetation is found [48]. At permanent sites, we found both water temperature and pH to have influence on *Bulinus* snail abundance. Snail density was observed to increase with rise in water temperature in permanent habitats. The reverse was observed with water pH with snail numbers observed to decrease with increase in water pH at permanent sites. Most previous studies found water temperature to be a more important predictor of snail numbers [49,50], but one study also found that snail abundance increased with a decrease in water pH [51].

Molecular techniques were used to detect and differentially identify *Schistosoma haematobium* and *Schistosoma bovis* parasites in *Bulinus* snails in this study. This technique was chosen because *S*. *haematobium* and *S*. *bovis* are both known to occur in The Gambia where they utilise the same intermediate host snails to accomplish their life processes [13]. Schistosomiasis transmission surveillance is usually complicated in areas where more than one schistosome species co-exist, and thus well robust species-specific detection techniques are required in such areas [24].

The study revealed that all three *Bulinus snails* collected in the study are involved in the transmission of at least one schistosome species. *Bulinus senegalensis*, the most widely distributed of all the *Bulinus* species in this study, was the only snail found infected with the human parasite, *Schistosoma haematobium*, albeit at just four study sites (3 in CRR and 1 in URR). These sites are in communities (Jahanka, Njoren Sare Jabel and Dingiri) that are known hotspots of urogenital schistosomiasis [7,30]. The rate of *S*. *haematobium* infection in *B*. *senegalensis* observed in this study corresponds to the prevalence of urogenital schistosomiasis infection observed in humans in these regions [6,7,30]. In recent studies on human populations, urogenital schistosomiasis was more common in CRR than URR. Previous studies conducted in The Gambia and other parts of West Africa also reported *B*. *senegalensis* as an intermediate host of *S*. *haematobium* [13,43,52]. In contrast, the bovine schistosome (*Schistosoma bovis*) had a much more widespread distribution occurring in 25 sites across CRR and URR, with all three *Bulinus* species found in this study involved in its transmission. It overlapped with *S*. *haematobium* at two of these sites. A lot of these sites are used by humans as well as livestock but are more frequented by livestock as was observed during the sample collection period. This finding is similar to what was recently observed in Ivory Coast [53] and Niger [54], where the livestock schistosome was found to be more common and occur in a wider geographical range compared to its human schistosome relative. Previous studies have also confirmed *B*. *senegalensis* as an intermediate host of *S*. *bovis* [13]. As *B*. *senegalensis* was found infected with both *S*. *haematobium* and *S*. *bovis* in this study, it might have the potential to harbour hybrids of these two species. *Bulinus forskalii* is also a confirmed intermediate host of the bovine schistosome in many parts of Africa [15,54], even though its role in the transmission of the human schistosome (*S*. *haematobium*) is not clear. One study implicated *B*. *forskalii* in the transmission of *S*. *haematobium* in Niger [55] but other studies undertaken in the same area disagreed with this finding, suggesting misidentification of *B*. *senegalensis* as *B*. *forskalii* and the notion that *B*. *forskalii* was not compatible with *S*. *haematobium* in the region [52,54]. Despite *B*. *truncatus* being the least common *Bulinus* species encountered in this study, it has the highest schistosome infection rate (12%) among the three *Bulinus* species and this shows how competent it is as an intermediate host of schistosomes. *Bulinus truncatus* is a proven intermediate host of both *S*. *haematobium* and *S*. *bovis* in several areas endemic for schistosomiasis [53,54,56,57]. It is also reported to harbour the hybrids of *S*. *haematobium* and *S*. *bovis* in many parts of West Africa [53,54,58]. The absence of *S*. *haematobium* infection in *B*. *truncatus* in this study is likely attributed to the low numbers of this snail in this study, with it found at just a few sites.

The existence of *Bulinus* snails infected with *S*. *haematobium* only in seasonal pools, confirms the important role of temporal rain-fed pools in the transmission of *S*. *haematobium* in Africa [43,45,52,57]. Conversely, *Bulinus* snails infected with *S*. *bovis* were recovered from all habitat types from which snails were collected in this study and previous studies found snails infected with *S*. *bovis* in several habitat types including seasonal pools, streams, swamp, ponds, ricefields, canals and dams [13,54,57].

The Gambia has initiated its national control programme for schistosomiasis and has reached several milestones of the programme targets. These include successful prevalence mapping of the disease and a mass drug administration campaign [29]. Subsequent interventions will include intermediate host snail control, sanitation and hygiene approaches which require knowledge on the intermediate host snails. The findings of this study provide new insight on the distribution and infection status of intermediate snail hosts of schistosomes in The Gambia which will be vital for the national schistosomiasis control initiative. The finding that *S*. *haematobium* transmission was observed at only four sites provides an opportunity for targeted interventions towards these communities. It also demonstrates the need for setting up a transmission surveillance system within the already established national schistosomiasis control programme, as snail sampling was undertaken at just a few time points in this study. Since transmission of schistosomiasis may vary from one time point to another, sustained sampling of snails will be required to better understand the transmission dynamics of *S*. *haematobium* in endemic areas of the country. The high incidence and wide geographical distribution of *S*. *bovis* in the study area serves as a reminder that this neglected ruminant schistosome needs to be given more attention in the study area, as the calls for a ‘one health approach’ to the control of schistosomiasis grow louder [17,34,57]. Additionally, intestinal schistosomiasis in livestock caused by *S*. *bovis* can result to potential economic loss through enteritis, emaciation and even death of livestock [16].

Although *Bulinus* snail sampling was conducted over a large geographical range across The Gambia, the sites were sampled during only four time points across two different years (2017 and 2019). More sustained sampling should be considered in future studies to better understand the snail-schistosome interactions at transmission sites in the country. Further studies are also needed to investigate hybridisation between *S*. *haematobium* group species as there is potential for *B*. *senegalensis* and *B*. *truncatus* to harbour hybrids in The Gambia [59].

## Supporting information

S1 Table Actual snail counts and GPS location coordinates of sampling sites. (DOCX)

S2 Table Physicochemical and environmental parameters. (DOCX)

S3 Table Rate of Infected *Bulinus* species per site. (DOCX)
