# Supplementary material for: Species diversity and distribution of schistosome intermediate snail hosts in The Gambia
Source: PLoS Negl Trop Dis. 2021 Oct 4;15(10):e0009823. doi: 10.1371/journal.pntd.0009823 (PMC8516291; doi:10.1371/journal.pntd.0009823)
Supplement: S1 Table — (DOCX) [file pntd.0009823.s001.docx]

**S1 Table. Actual snail counts and GPS location coordinates of sampling sites.**

| **No** | **Site** | **GPS**  **coordinates** | **Region** | **Habitat**  **type** | **Snail abundance** | | | |  |
| --- | --- | --- | --- | --- | --- | --- | --- | --- | --- |
|  |  |  |  |  | ***B. senegalensis*** | ***B. forskalii*** | ***B.***  ***truncatus*** | **Total**  ***Bulinus*** | **Other snails** |
| 1 | Sare Bolli | 13.609017  -15.14675 | CRR | Seasonal  pool | 12 | 13 | 0 | **25** | *Lanistes spp*: 5 |
| 2 | Madina  Nfally 1 | 13.543967  -14.959617 | CRR | Stream | 0 | 0 | 10 | **10** |  |
| 3 | Madina  Nfally 2 | 13.54395  -14.959117 | CRR | Seasonal  pool | 110 | 0 | 0 | **110** |  |
| 4 | Kudang  Bridge | 13.658867  -15.071667 | CRR | Seasonal  pool | 1 | 0 | 0 | **1** |  |
| 5 | Kundang  Tenda | 13.6891  -15.077283 | CRR | River | 0 | 0 | 0 | **0** |  |
| 6 | Jarreng  Bridge | 13.617917  -15.196067 | CRR | Stream | 0 | 0 | 0 | **0** |  |
| 7 | Jarreng  Badala | 13.6396  -15.19395 | CRR | River | 0 | 0 | 0 | **0** |  |
| 8 | Sare Anis 1 (sofaniama Bolong) | 13.556167  -15.188367 | CRR | Stream | 0 | 0 | 0 | **0** |  |
| 9 | Sare Anis 2 | 13.558167  -15.1867 | CRR | Stream | 0 | 0 | 0 | **0** |  |
| 10 | Daru | 13.36944  -14.60019 | CRR | Seasonal  pool | 161 | 0 | 0 | **161** |  |
| 11 | Sanka  Bari | 13.34949  -14.63018 | CRR | Seasonal  pool | 115 | 0 | 0 | **115** |  |
| 12 | Njoren | 13.34598  -14.64303 | CRR | Seasonal  pool | 119 | 0 | 0 | **119** |  |
| 13 | Sinchu  Bokar | 13.41695  -14.70525 | CRR | Seasonal  pool | 83 | 0 | 0 | **83** |  |
| 14 | Sare Madi  Ganteh | 13.41991  -14.72219 | CRR | Seasonal  pool | 91 | 0 | 0 | **91** |  |
| 15 | Kerr Ousman Boye | 13.41423  -14.74174 | CRR | Seasonal  pool | 70 | 0 | 0 | **70** |  |
| 16 | Sare Jabel | 13.38333  -14.70000 | CRR | Seasonal  pool | 238 | 0 | 0 | **238** |  |
| 17 | Jahanka | 13.37776  -14.72773 | CRR | Seasonal  pool | 68 | 0 | 0 | **68** |  |
| 18 | Sare Madi  Babadi | 13.44930  -14.69858 | CRR | Seasonal  pool | 17 | 0 | 0 | **17** |  |
| 19 | Sare Chewto | 13.42820  -14.59223 | CRR | Seasonal  pool | 44 | 16 | 0 | **60** |  |
| 20 | Bansang | 13.42670  -14.65623 | CRR | Rice field | 0 | 76 | 0 | **76** |  |
| 21 | Sololo | 13.46059  -14.68370 | CRR | Swamp | 5 | 20 | 0 | **25** |  |
| 22 | Dobo | 13.47782  -14.64302 | CRR | Seasonal  pool | 39 | 0 | 0 | **39** |  |
| 23 | Dembakally | 13.59259  -14.61033 | CRR | Seasonal  pool | 2 | 0 | 0 | **2** |  |
| 24 | Changai 2 | 13.58624  -14.67834 | CRR | Seasonal  pool | 92 | 0 | 0 | **92** |  |
| 25 | Changai 1 | 13.57868  -14.66852 | CRR | Seasonal pool | 0 | 0 | 0 | **0** |  |
| 26 | Raneru 2 | 13.63299  -14.54088 | CRR | Seasonal pool | 1 | 0 | 0 | **1** |  |
| 27 | Raneru 1 | 13.63092  -14.54611 | CRR | Seasonal pool | 0 | 0 | 0 | **0** |  |
| 28 | Kuntaur Fulakunda/  Jakaba | 13.65983  -14.87867 | CRR | Rice field | 0 | 24 | 0 | **24** |  |
| 29 | Jokul  Ndowen | 13.78485  -14.88732 | CRR | Seasonal pool | 0 | 0 | 0 | **0** |  |
| 30 | Pallol 2 | 13.75574  -14.85203 | CRR | Seasonal pool | 0 | 0 | 0 | **0** |  |
| 31 | Pallol 1 | 13.76528  -14.85914 | CRR | Seasonal pool | 0 | 0 | 0 | **0** |  |
| 32 | Sapu | 13.552733  -14.89715 | CRR | Rice field | 0 | 0 | 0 | **0** |  |
| 33 | Pacharr 1 | 13.517733  -14.8543 | CRR | Irrigation canal | 0 | 12 | 41 | **53** | *Radix natalensis*:  57; *Gyralus spp*: 4 |
| 34 | Pacharr 2 | 13.522917  -14.856383 | CRR | Rice field | 0 | 10 | 0 | **10** | *Gyralus spp*: 32 |
| 35 | Dalaba | 13.598217  -15.21305 | CRR | Stream | 2 | 18 | 0 | **20** | *Gyralus spp*: 27 |
| 36 | Kuntaur  Wharf Town | 13.676867  -14.885517 | CRR | Rice field | 0 | 18 | 0 | **18** | *Radix natalensis*:  1;  *Gyralus spp*: 7;  *Lanistes spp*: 2 |
| 37 | Pallang | 13.66535  -14.86895 | CRR | Stream | 0 | 0 | 0 | **0** | *Radix natalensis*:  107 |
| 38 | Sankulay  Kunda | 13.51318  -14.76011 | CRR | Seasonal pool | 26 | 0 | 0 | **26** |  |
| 39 | Janjanbureh | 13.52557  -14.7639 | CRR | Rice field | 7 | 12 | 0 | **19** |  |
| 40 | Kuntaur 2 | 13.66493  -14.89007 | CRR | Rice field | 0 | 0 | 0 | **0** |  |
| 41 | Wassu | 13.69895  -14.88602 | CRR | Seasonal pool | 36 | 0 | 0 | **36** |  |
| 42 | Pacharr 3 | 13.49936  -14.84492 | CRR | Seasonal pool | 0 | 0 | 0 | **0** |  |
| 43 | Jahally | 13.54481  -14.9659 | CRR | Seasonal pool | 27 | 0 | 0 | **27** |  |
| 44 | Choya | 13.54108  -15.24912 | CRR | Stream | 0 | 31 | 0 | **31** |  |
| 45 | Cha Kunda | 13.38555  -14.53051 | CRR | Seasonal pool | 0 | 0 | 0 | **0** |  |
| 46 | Sare Modou | 13.39387  -14.61993 | CRR | Seasonal pool | 0 | 0 | 0 | **0** |  |
| 47 | Dobo 2 | 13.44479  -14.64437 | CRR | Seasonal pool | 6 | 0 | 0 | **6** |  |
| 48 | Kunting | 13.53171  -14.66579 | CRR | Seasonal pool | 40 | 0 | 0 | **40** |  |
| 49 | Sami  Karantaba | 13.55906  -14.56813 | CRR | Seasonal pool | 1 | 0 | 0 | **1** |  |
| 50 | Dembakally Njagga | 13.59446  -14.59227 | CRR | Seasonal pool | 0 | 0 | 0 | **0** |  |
| 51 | Dingiri 1  (Dingiri Kore) | 13.28580  -14.04369 | URR | Seasonal pool | 81 | 0 | 0 | **81** |  |
| 52 | Dingiri 2 | 13.30101  -14.05310 | URR | Seasonal pool | 0 | 0 | 0 | **0** |  |
| 53 | Kuwonku  (Koilu Dalo) | 13.42475  -14.33871 | URR | Seasonal pool | 96 | 0 | 0 | **96** |  |
| 54 | Touba  Woppa | 13.45798  -14.226 | URR | Seasonal pool | 0 | 0 | 0 | **0** |  |
| 55 | Madina  Samaco (Toro) | 13.29197  -14.01417 | URR | Seasonal pool | 68 | 0 | 0 | **68** |  |
| 56 | Bajakunda | 13.47509  -14.04987 | URR | Seasonal pool | 30 | 0 | 0 | **30** |  |
| 57 | Koli Bantang (bamba dala) | 13.43636  -14.17679 | URR | Seasonal pool | 33 | 0 | 0 | **33** |  |
| 58 | Murreh Kunda | 13.53689  -14.0881 | URR | Seasonal pool | 0 | 0 | 0 | **0** |  |
| 59 | Diabugu  Basilla (misigi) | 13.316533  -13.979717 | URR | Seasonal pool | 201 | 0 | 0 | **201** |  |
| 60 | Kisskiss | 13.30309  -14.16010 | URR | Stream | 0 | 0 | 1 | **1** |  |
| 61 | Basse  Kobakunda | 13.32553  -14.22385 | URR | Stream | 0 | 10 | 0 | **10** |  |
| 62 | Demba  Kunda | 13.25185  -14.27178 | URR | Stream | 0 | 20 | 0 | **20** |  |
| 63 | Dampha  Kunda | 13.32033  -14.20051 | URR | Stream | 0 | 28 | 0 | **28** | *Gyralus spp*: 2 |
| 64 | Chamoi | 15.55500  -14.17032 | URR | Stream | 0 | 16 | 0 | **16** | *Gyralus spp*: 5 |
| 65 | Sare Alpha | 13.36609  -13.98098 | URR | Stream | 0 | 3 | 0 | **3** | *Gyralus spp*: 1 |
| 66 | Sare Alpha2 | 13.364717  -13.976367 | URR | Stream | 0 | 0 | 0 | **0** |  |
| 67 | Missira Ba Mariama | 13.33295  -13.94445 | URR | Stream | 0 | 77 | 39 | **116** | *Gyralus spp*: 33 |
| 68 | Sotuma Sire/Samba | 13.29975  -14.312933 | URR | Stream | 0 | 157 | 0 | **157** | *Gyralus spp*: 9;  *Lanistes spp*: 1 |
| 69 | Sutukonding | 13.37796  -14.22345 | URR | Seasonal pool | 69 | 0 | 0 | **69** |  |
| 70 | Kanubeh | 13.3208  -14.2964 | URR | Seasonal pool | 59 | 0 | 0 | **59** |  |
| 71 | Hellakunda | 13.27951  -14.37461 | URR | Stream | 0 | 2 | 0 | **2** |  |
| 72 | Madina  Samba Jawo | 13.32174  -14.42531 | URR | Seasonal pool | 1 | 0 | 0 | **1** |  |
| 73 | Mankamang Kunda | 13.3267  -14.45029 | URR | Seasonal pool | 0 | 0 | 0 | **0** |  |
| 74 | Sare Pateh Bakery | 13.32705  -14.51422 | URR | Seasonal pool | 16 | 0 | 0 | **16** |  |
| 75 | Darsilameh | 13.40236  -14.26843 | URR | Seasonal pool | 0 | 0 | 0 | **0** |  |
| 76 | Sumakunda | 13.39877  -14.3152 | URR | Seasonal pool | 0 | 0 | 0 | **0** |  |
| 77 | Sare Fodike | 13.39596  -14.35416 | URR | Seasonal pool | 0 | 0 | 0 | **0** |  |
| 78 | Diabug  Batapa | 13.39236  -14.41309 | URR | Seasonal pool | 0 | 0 | 0 | **0** |  |
| 79 | Kuwonku 2 | 13.39877  -14.3152 | URR | Seasonal pool | 0 | 0 | 0 | **0** |  |
| 80 | Chamoi 2 | 13.46208  -14.10999 | URR | Seasonal pool | 17 | 0 | 0 | **17** |  |
| 81 | Sutukoba | 13.49646  -14.02709 | URR | Seasonal pool | 0 | 0 | 0 | **0** |  |
| 82 | Makka  Masireh | 13.56516  -13.99013 | URR | Seasonal pool | 0 | 0 | 0 | **0** |  |
| 83 | Wellingara  Yarreh | 13.54152  -14.00332 | URR | Seasonal pool | 43 | 0 | 0 | **43** |  |
| 84 | Wellingara  Yarreh 2 | 13.54845  -13.99094 | URR | Seasonal pool | 0 | 0 | 0 | **0** |  |
| 85 | Gunjur | 13.55409  -14.02202 | URR | Seasonal pool | 0 | 0 | 0 | **0** |  |
| 86 | Murreh  Kunda 2 | 13.54345  -14.05137 | URR | Seasonal pool | 0 | 0 | 0 | **0** |  |
| 87 | Sare Juldeh | 13.33624  -14.04408 | URR | Seasonal pool | 0 | 0 | 0 | **0** |  |
| 88 | Suduwol | 13.37747  -13.97064 | URR | Seasonal pool | 27 | 0 | 0 | **27** |  |
| 89 | Kusan/  Temato | 13.38461  -13.93328 | URR | Seasonal pool | 0 | 0 | 0 | **0** |  |
| 90 | Basse Kabakamma | 13.30485  -14.20964 | URR | Stream | 0 | 20 | 0 | **20** |  |
| 91 | Kanjapat | 13.22957  -15.9194 | WR | Seasonal pool | 0 | 0 | 0 | **0** |  |
| 92 | Ndemban | 13.20117  -16.34642 | WR | Swamp | 1 | 19 | 0 | **20** |  |
| 93 | Kampanti | 13.19921  -16.11379 | WR | Seasonal pool | 0 | 0 | 0 | **0** |  |
| 94 | Kafuta | 13.19351  -16.44991 | WR | Seasonal pool | 0 | 0 | 0 | **0** |  |
| 95 | Jiboro | 13.1678  -16.57662 | WR | Swamp | 0 | 0 | 0 | **0** |  |
| 96 | Sifoe/Kitty | 13.21601  -16.67436 | WR | Seasonal pool | 28 | 0 | 0 | **28** |  |
| 97 | Kotu | 13.460706  -16.69386 | WR | Swamp | 0 | 0 | 0 | **0** |  |
| 98 | Wellengara  Ba 1 | 13.414983  -15.242017 | LRR | Seasonal pool | 1 | 0 | 0 | **1** |  |
| 99 | Wellengara  Ba 2 | 13.391717  -15.277667 | LRR | Stream | 0 | 0 | 0 | **0** |  |
| 100 | Dongoro Ba | 13.381733  -15.302017 | LRR | Stream | 0 | 0 | 0 | **0** |  |
| 101 | Nioro Jataba | 13.42922  -15.43882 | LRR | Seasonal pool | 0 | 0 | 0 | **0** |  |
| 102 | Kiang  Bambako 1 | 13.40347  -15.77289 | LRR | Seasonal pool | 0 | 0 | 0 | **0** |  |
| 103 | Kiang  Bambako 2 | 13.40349  -15.77303 | LRR | Rice field | 0 | 0 | 0 | **0** |  |
| 104 | Kolior | 13.4104  -15.67651 | LRR | Seasonal pool | 0 | 0 | 0 | **0** |  |
| 105 | Jarra Buiba | 13.43324  -15.44416 | LRR | Seasonal pool | 0 | 0 | 0 | **0** |  |
| 106 | Jenoi | 13.49045  -15.56171 | LRR | Swamp | 0 | 0 | 0 | **0** |  |
| 107 | Farafenni 1 | 13.55827  -15.58444 | NBR | Rice field | 0 | 0 | 0 | **0** |  |
| 108 | Farafenni 2 | 13.57205  -15.61475 | NBR | Seasonal pool | 0 | 0 | 0 | **0** |  |
| 109 | Bangally | 13.50906  -16.41188 | NBR | Seasonal pool | 0 | 0 | 0 | **0** |  |
| 110 | Ndungu  Kebbeh | 13.53477  -16.32685 | NBR | Seasonal pool | 0 | 0 | 0 | **0** |  |
| 111 | Kerr Selleh | 13.53314  -16.28884 | NBR | Seasonal pool | 0 | 0 | 0 | **0** |  |
| 112 | Njama  Sinyan | 13.53407  -16.26364 | NBR | Seasonal pool | 0 | 0 | 0 | **0** |  |
| 113 | Jamagen | 13.53238  -16.24413 | NBR | Seasonal pool | 0 | 0 | 0 | **0** |  |
| 114 | Berending | 13.4835  -16.46419 | NBR | Stream | 0 | 0 | 0 | **0** |  |
| **Total** |  |  |  |  | **2184** | **602** | **91** | **2877** |  |
