# Supplementary material for: Species diversity and distribution of schistosome intermediate snail hosts in The Gambia
Source: PLoS Negl Trop Dis. 2021 Oct 4;15(10):e0009823. doi: 10.1371/journal.pntd.0009823 (PMC8516291; doi:10.1371/journal.pntd.0009823)
Supplement: S2 Table — (DOCX) [file pntd.0009823.s002.docx]

**S2 Table. Physicochemical and environmental parameters**

| **Habitat type** | **Site name** | **Aquatic vegetation**  **present** | **Algae**  **present** | **Water temp.** | | **Water pH** | |
| --- | --- | --- | --- | --- | --- | --- | --- |
| Seasonal | Sare Bolli/Bamba Kolong | Yes | No | 23.2 | Range: 22.1⁰C –38.3⁰C  Median: 29.3⁰C  Interquartile range: 27.8⁰C – 33.1⁰C | 7.1 | Range: 6.42 – 8.16  Median: 6.95  Interquartile range: 6.78 – 7.26 |
|  | Daru | Yes | No | 27.8 |  | 6.42 |  |
|  | Njoren/Sanka Bari Pool 1 | No | No | 29.3 |  | 6.62 |  |
|  | Njoren/Sanka Bari Pool 2 | Yes | Yes | 30.1 |  | 7.26 |  |
|  | Sinchu Bokar | Yes | No | 33.3 |  | 6.78 |  |
|  | Sare Madi Ganteh | Yes | No | 35.9 |  | 6.79 |  |
|  | Kerr Ousman Boye | yes | Yes | 33.1 |  | 6.95 |  |
|  | Sare Jabel | yes | No | 28.9 |  | 6.91 |  |
|  | Jahanka | yes | No | 31.4 |  | 6.67 |  |
|  | Sare Madi Babadi | No | No | 36.4 |  | 6.67 |  |
|  | Sare Chewto | Yes | No | 29.1 |  | 7.19 |  |
|  | Dobo | No | No | 34.9 |  | 6.99 |  |
|  | Dembakally | No | No | 31.5 |  | 7.24 |  |
|  | Changai Pool 2 | Yes | No | 26.5 |  | 7.64 |  |
|  | Raneru Pool 2 | Yes | No | 34.6 |  | 6.87 |  |
|  | Jokul Ndowen | No | No | 32.3 |  | 7.53 |  |
|  | Pallol Pool 2 | No | No | 27.2 |  | 7.05 |  |
|  | Dingiri pool 1 (Dingiri Kore) | yes | No | 28.3 |  | 6.91 |  |
|  | Dingiri pool 2 | No | No | 29.5 |  | 6.95 |  |
|  | Kuwonku (Koilu Dalo) | Yes | No | 22.1 |  | 6.91 |  |
|  | Madina Samaco (Toro) | Yes | No | 28.3 |  | 6.67 |  |
|  | Bajakunda | yes | yes | 24.4 |  | 6.7 |  |
|  | Koli Bantang (bamba dala) | No | No | 38.3 |  | 7.54 |  |
|  | Diabugu Basilla (misigi) | yes | No | 28.3 |  | 7.44 |  |
|  | Wellengara Ba 1 | No | No | 30.5 |  | 7.84 |  |
|  | Kudang Bridge | yes | No | 28.3 |  | 7.05 |  |
| Permanent | Madina Nfally pool 1 | Yes | No | 25.6 | Range: 23.1⁰C – 33.9⁰C  Median: 27.6⁰C  Interquartile range: 25.3⁰C – 30.0⁰C | 6.63 | Range: 5.52 – 7.55  Median: 6.96  Interquartile range: 6.37 – 7.17 |
|  | Madina Nfally pool 2 | No | No | 24.5 |  | 8.16 |  |
|  | Kundang Tenda | No | No | 23.2 |  | 7.55 |  |
|  | Jarreng Bridge/Bolong | yes | No | 27.3 |  | 7.18 |  |
|  | Jarreng Badala | No | No | 27.9 |  | 7.49 |  |
|  | Sare Anis (sofaniama Bolong) | Yes | No | 28.3 |  | 7.16 |  |
|  | Sare Anis Pool | Yes | Yes | 23.1 |  | 7.13 |  |
|  | Bansang Rice field | yes | No | 30.5 |  | 6.73 |  |
|  | Sololo | yes | No | 33.9 |  | 5.52 |  |
|  | Kuntaur Fulakunda/Jakaba | yes | No | 26.6 |  | 6.94 |  |
|  | Kisskiss | yes | No | 25.1 |  | 6.38 |  |
|  | Basse Kobakunda | yes | No | 25.5 |  | 6.17 |  |
|  | Demba Kunda | Yes | No | 23.3 |  | 6.34 |  |
|  | Dampha Kunda | Yes | No | 25.3 |  | 6.33 |  |
|  | Chamoi | Yes | Yes | 30 |  | 7.09 |  |
|  | Sare Alpha | Yes | No | 24.8 |  | 6.97 |  |
|  | Suduwol Sare Alpha Bridge/Bolong | Yes | No | 24.3 |  | 6.99 |  |
|  | Sapu | yes | No | 26.9 |  | 7.37 |  |
|  | Pacharr 1 | yes | No | 28.1 |  | 6.69 |  |
|  | Pacharr 2 | yes | yes | 28.2 |  | 7.13 |  |
|  | Dalaba | Yes | Yes | 30.1 |  | 6.5 |  |
|  | Kuntaur Wharf Town | yes | No | 33 |  | 6.98 |  |
|  | Pallang | Yes | Yes | 31 |  | 6.8 |  |
|  | Missira Ba Mariama | Yes | Yes | 31.1 |  | 5.97 |  |
|  | Sotuma Sire/Samba | Yes | Yes | 27.9 |  | 5.99 |  |
|  | Wellengara Ba 2 | Yes | No | 27.7 |  | 7.52 |  |
|  | Dongoro Ba | No | No | 27.4 |  | 7.35 |  |
