# Supplementary material for: Species diversity and distribution of schistosome intermediate snail hosts in The Gambia
Source: PLoS Negl Trop Dis. 2021 Oct 4;15(10):e0009823. doi: 10.1371/journal.pntd.0009823 (PMC8516291; doi:10.1371/journal.pntd.0009823)
Supplement: S3 Table — (DOCX) [file pntd.0009823.s003.docx]

**S3 Table. Rate of Infected *Bulinus* species per site**

| **No.** | **Site** | **Region** | ***Bulinus* species** | **No. Tested** | **No. Infected (rate)** | |
| --- | --- | --- | --- | --- | --- | --- |
|  |  |  |  |  | ***S. haematobium*** | ***S. bovis*** |
| 1 | Sare Bolli | CRR | *B. senegalensis* | 10 | 0 | 0 |
|  |  |  | *B. forskalii* | 11 | 0 | 0 |
| 2 | Madina Nfally 1 | CRR | *B. truncatus* | 10 | 0 | 1 (10%) |
| 3 | Madina Nfally 2 | CRR | *B. senegalensis* | 20 | 0 | 6 (30%) |
| 4 | Kudang Bridge | CRR | *B. senegalensis* | 1 | 0 | 0 |
| 5 | Njoren | CRR | *B. senegalensis* | 20 | 1 (5%) | 0 |
| 6 | Daru | CRR | *B. senegalensis* | 20 | 0 | 1 (5%) |
| 7 | Sanka Bari | CRR | *B. senegalensis* | 20 | 0 | 0 |
| 8 | Sinchu Bokary | CRR | *B. senegalensis* | 20 | 0 | 2 (10%) |
| 9 | Sare Madi Ganteh | CRR | *B. senegalensis* | 20 | 0 | 0 |
| 10 | Kerr Ousman Boye | CRR | *B. senegalensis* | 20 | 0 | 8 (40%) |
| 11 | Sare Jabel | CRR | *B. senegalensis* | 20 | 1 (5%) | 5 (25%) |
| 12 | Jahanka | CRR | *B. senegalensis* | 20 | 3 (15%) | 0 |
| 13 | Sare Madi Babadi | CRR | *B. senegalensis* | 17 | 0 | 0 |
| 14 | Sare Chewto | CRR | *B. senegalensis* | 20 | 0 | 0 |
|  |  |  | *B. forskalii* | 10 | 0 | 0 |
| 15 | Bansang | CRR | *B. forskalii* | 20 | 0 | 0 |
| 16 | Sololo | CRR | *B. senegalensis* | 5 | 0 | 0 |
|  |  |  | *B. forskalii* | 20 | 0 | 2 (10%) |
| 17 | Dobo | CRR | *B. senegalensis* | 20 | 0 | 1 (5%) |
| 18 | Dembakally | CRR | *B. senegalensis* | 2 | 0 | 0 |
| 19 | Changai 2 | CRR | *B. senegalensis* | 20 | 0 | 1 (5%) |
| 20 | Kuntaur Fulakunda/Jakaba | CRR | *B. forskalii* | 20 | 0 | 0 |
| 21 | Pacharr 1 | CRR | *B. forskalii* | 12 | 0 | 3 (25%) |
|  |  |  | *B. truncatus* | 20 | 0 | 2 (10%) |
| 22 | Pacharr 2 | CRR | *B. forskalii* | 10 | 0 | 0 |
| 23 | Dalaba | CRR | *B. forskalii* | 17 | 0 | 1 (5.9%) |
| 24 | Kuntaur Wharf Town | CRR | *B. forskalii* | 15 | 0 | 6 (40%) |
| 25 | Sankulay Kunda | CRR | *B. senegalensis* | 19 | 0 | 1(5.3%) |
| 26 | Janjanbureh | CRR | *B. senegalensis* | 6 | 0 | 0 |
|  |  |  | *B. forskalii* | 10 | 0 | 0 |
| 27 | Wassu | CRR | *B. senegalensis* | 20 | 0 | 0 |
| 28 | Jahally | CRR | *B. senegalensis* | 20 | 0 | 2(10%) |
| 29 | Choya | CRR | *B. forskalii* | 20 | 0 | 0 |
| 30 | kunting | CRR | *B. senegalensis* | 20 | 0 | 0 |
| 31 | Dingiri 1 | URR | *B. senegalensis* | 20 | 2 (10) | 2 (10%) |
| 32 | Kuwonku | URR | *B. senegalensis* | 20 | 0 | 3 (15%) |
| 33 | Madina Samaco | URR | *B. senegalensis* | 20 | 0 | 1 (5%) |
| 34 | Bajakunda | URR | *B. senegalensis* | 20 | 0 | 4 (20%) |
| 35 | kolibantang | URR | *B. senegalensis* | 20 | 0 | 1 (5%) |
| 36 | Diabugu Basilla | URR | *B. senegalensis* | 20 | 0 | 4 (20%) |
| 37 | Demba Kunda | URR | *B. forskalii* | 11 | 0 | 1 (9.1%) |
| 38 | Misra Ba Mariama | URR | *B. forskalii* | 20 | 0 | 2 (10%) |
|  |  |  | *B. truncatus* | 20 | 0 | 3 (15%) |
| 39 | Sotuma Sire/Samba | URR | *B. forskalii* | 20 | 0 | 4 (20%) |
| 40 | Sutukonding | URR | *B. senegalensis* | 20 | 0 | 0 |
| 41 | Kanubeh | URR | *B. senegalensis* | 20 | 0 | 2 (10%) |
| 42 | Madina Samba Jawo | URR | *B. senegalensis* | 1 | 0 | 0 |
| 43 | Sare Pateh Bakery | URR | *B. senegalensis* | 12 | 0 | 0 |
| 44 | Wellingara Yarreh | URR | *B. senegalensis* | 20 | 0 | 1 (5%) |
| 45 | Suduwol | URR | *B. senegalensis* | 20 | 0 | 0 |
| 46 | Basse Kabakama | URR | *B. forskalii* | 20 | 0 | 0 |
| 47 | Ndemban | WR | *B. senegalensis* | 1 | 0 | 0 |
|  |  |  | *B. forskalii* | 11 | 0 | 0 |
| 48 | Siffoe/Kitty | WR | *B. senegalensis* | 12 | 0 | 0 |
| Total | | | | 883 | 7 (0.8) | 70 (7.9) |
